# Bone Glue Modified Asphalt: A Step towards Energy Conservation and Environment Friendly Modified Asphalts

**DOI:** 10.1155/2014/807043

**Published:** 2014-09-29

**Authors:** Hashim Raza Rizvi, Mohammad Jamal Khattak, August A. Gallo

**Affiliations:** Department of Civil Engineering, University of Louisiana at Lafayette, USA

## Abstract

Asphalt has been modified for the past several decades using various additives, including synthetic polymers. Polymer modification improves structural and engineering characteristics of the binder, which is a result of improvement in rheological characteristics of binder as well as its adhesion capability with the aggregate. Such enhancement inevitably enhances the performance characteristics of hot mix asphalts (HMA) such as fatigue life, resistance to rutting, and thermal cracking. Even though polymer-modified HMA is popular in North America and European countries, its use is still limited in developing countries of Southeast Asia due to high costs associated with its manufacturing, processing, and energy consumption. In this study, a new kind of asphalt modifier derived from animal wastes, such as bones, hides, and flesh commonly known as Bone Glue, is studied. This biomaterial which is a by-product of food and cattle industries is cheap, conveniently available, and produced locally in developing countries. The results of the research study showed that the bone glue can easily be mixed with asphalt without significantly altering the asphalt binder's viscosity and mixing and compaction temperatures of HMA. Additionally, improvements in complex shear modulus for a range of temperatures were also determined and it was found that complex shear modulus was improved by bone glue modification.

## 1. Introduction and Background

Asphalt modification is a common practice since the last two decades throughout the United States of America (USA). Asphalt modification with synthetic and natural polymers is not a new concept in this industry, as one can find traces of modification back in 1843 [1]. Initially, in Europe many projects of asphalt modification were underway in the 1930s [2]. Synthetic rubber (neoprene) was used for asphalt modification in Latin America in the 1950s [3]. Europe was ahead of USA in 1970s as the contractor in Europe preferred to afford the initial cost instead of bearing the maintenance cost [2]. The disadvantage of higher initial cost of polymer-modified asphalt limited its use in US [4]. However, with the invention of newer polymers, European technologies began to be used in USA [5, 6]. According to a survey in 1997, 47 out of 50 states reported that they would be using modified asphalt in the future and 35 of the states from the 47 reported that they would use greater amounts [7]. This inclination towards the use of modified asphalt was due to its proven advantages over the lifetime of the pavements. There were many types of polymers added to the asphalt in order to modify it. Some of them are SBS (styrene-butadiene-styrene), SBR (styrene-butadiene-rubber), Elvaloy rubber, EVA (ethylene-vinyle acetate), HDP (high density polyethylene), LDP (low density polyethylene), tire rubber, and others. This polymer-modified asphalt was expected to show greater elastic recovery, a higher softening point, greater viscosity, greater cohesive strength, and greater ductility [3, 8]. It was found that polymer modification can improve structural and engineering properties of the binder, which is a result of improvement in rheological characteristics of binder as well as its adhesion capability with the aggregate [9]. Enhancement in these characteristics inevitably enhanced the performance characteristics of asphalt, that is, fatigue life, resistance to rutting, and thermal cracking.

Polymer-modified asphalt is being used frequently in almost every part of the world, especially in developed countries. The amount of energy used for the modification and price of the polymer is a big question mark for the researchers and contractors. Polymers need high temperatures (150°F to 375°F) and an extended period of time (60 to 200 min) to achieve a homogenous blend with asphalt. Moreover, it is not about preparation of modified asphalt; yet whether it is sustainable enough to resist the environmental conditions or not is also a big concern. Last but not least, HMA resistance to the cutbacks (Diesel, Gasoline, Engine oil etc.). These concerns are very important to consider while modifying asphalt. Since polymer modification of asphalt is expensive, especially in developing countries, therefore its use is very limited in developing countries.

Most commonly used asphalt modifier is SBS polymer. The price of SBS dramatically increased due to shortage of styrene-butadiene polymers in the asphalt industry since 2008. Asphalt costs approximately $0.6 per kg, whereas recycled tire rubber costs about $0.3 per kg. Likewise, polymer may cost more than $1 per kg (e.g., SBS price is around $1.25 per kg). The shortage involved a variety of polymers, including linear and radial SBS polymers and diblock SBS polymers. The main reason why SBS is short in supply is its raw material, which is “ethylene.” Alternatives of SBS are GTR (ground tire rubber), which requires high temperature and very high shear mixing conditions. Chemical stabilizers are also added in the mixing process, which increase the cost of the material as well. Some other alternatives are SBR-latex, EVA (ethyle vinyl acitate), and PPA (polyphosphoric acid). SBR-latex is not storage stable, EVA is used in warm climates, and PPA is merely an extender; it is not an alternative [10]. As far as tire rubber is concerned, it requires almost 340°F to 410°F temperature to blend the rubber in the asphalt. This is very high temperature for asphalt blending, especially at 3500 rpm.

In this study, an attempt has been made to utilize “bone glue” (BG), a by-product of food and cattle industries, to modify asphalt binders. BG is protein-based glue made from collagen extracted from animal bones, hides, and flesh waste. Collagen is a group of naturally occurring proteins. It is the most abundant protein in animals that makes up 25% to 35% of the whole body protein content. It has some types, out of which collagen I is the main type of collagen, which is 90% of the total collagen present in the body. This type of collagen is acquired from skin, organs, bones, and so forth. Raw material for BG is animal waste. It is easily available in developing countries; however the production of this product is very limited due to its limited use, mainly in local furniture industry. If this product is encouraged, the price can be reduced as well, which will inevitably reduce the initial cost. Presently, it is about $0.8 to $1.9 per kg as per manufacturer in Pakistan, which can be further reduced if purchased in bulk as tons. A quick survey of this industry, conducted by the researchers in July 2013, revealed that approximately 20 factories are currently producing this product all over Pakistan. The limited use of the product reduces the number of producers to such a low level. Advent of synthetic glue also played its role in limiting the use of bone glue. It can be determined through this survey that if demand increases, the supply will also increase, which will inevitably reduce the cost as well. Apart from the above discussion, the current price of BG is easily comparable with any other polymer available in the market. In many cases, especially SBS, SBR, and Elvaloy, the price of BG is still as low as 50% of the present cost of other polymers.

A thorough and extensive literature review revealed that no research has been conducted on the use of BG for asphalt modification till now. The reason for selecting BG for modification is three-fold: it is cheap and conveniently and locally available for developing countries who have been manufacturing this product for centuries, and the process of modification is environment friendly. Cost concern is the main reason for not using modified asphalt as these countries cannot afford the manufacturing, processing, and construction costs of polymer-modified asphalt. However, they need to improve the performance of the pavements in particular at high temperature ranges. It is believed that the BG can be a viable and useful alternative for asphalt modification.

## 2. Objectives of Study

The objectives of study are as follows.

(1) Explore the best possible method to blend BG in asphalt binder based on least possible energy and time consumption.
(2) Evaluate the viscosity and *G* ^*^ of neat and BG-modified binders at various temperatures and load frequency.

## 3. Test Materials and Methods

### 3.1. Materials

Two viscosity graded asphalts, AC 5 (PG58-22) and PAC 30 (PG70-22), were modified with 2.5, 5, 10, and 20 percent of BG. The BG, in pallets, was supplied by a vender from Pakistan.

### 3.2. Mixing Process

In order to achieve homogeneous mix, it is very important to evolve a thorough, manageable, and low cost process. The idea behind the research is to develop a material, which is strong, sustainable, and resistant to the distress; however, it is not advisable to come up with a procedure, which is highly expensive or significantly dangerous and damaging.

The problem with BG is its inability to melt at high temperatures. When it is heated it burns and turns to ash. This inability of melting was a considerable issue in asphalt mixing, as asphalts are always mixed at high temperatures with aggregates in order to achieve homogeneous mixing. It was discovered that BG is soluble in water. Even at room temperatures, BG dissolves in water if kept for longer time. However, if water is heated, this process of dissolution can be significantly expedited. Hence, it was decided that, at first stage, dissolve the BG in water and then the solution of water and BG would be mixed in asphalt binder, since water can easily be evaporated at temperature range of 115°C to 135°C, which is also least damaging to the binder. In order to organize the mixing time, temperature, and quantity, 25 different mixtures were prepared. It was found that the following procedure was quick and energy conserving.

In order to mix 10% of BG, by weight of asphalt binder, 20 gm of BG pallets was put in 40 gm of water at room temperature. The dry weight of the pan, weight with water, and BG were recorded. The asphalt binder was put in preheated oven at 135°C for one hour. Time was set for 50 minutes in order to make solution of BG and water. After 50 minutes, the pan with water and BG was weighed again. After weighing, the pan was heated on a burner and continuously stirred for 10 minutes. BG dissolves in water very quickly, while heating. The moment water starts boiling the burner is slowed down in order to avoid spillage. When the solution becomes homogenous (visually), the pan and solution are weighed again. Required amount of asphalt binder is taken in a beaker of 600 mL and the BG-solution is added to the binder. The weights of beaker with binder, solution, and spindle are recorded. The beaker is put in an oil bath for mixing at 130°C using shear mixer at 1000 rpm. The beaker is not covered from top in order to let the water evaporate. The mixing was stopped after 70 minutes or with respect to the percentage of water. The weight of beaker with mixture and spindle is recorded again. Mass loss due to evaporation was determined. When the mass loss equals the actual mass of the water added, the mixing time was considered adequate.

### 3.3. Rotational Viscosity

Viscosity is key information for asphalt mixtures as HMA mixing and compaction depend on the values determined through viscosity testing. The superpave mix design recommends rotational viscometer [11] for determining the viscosity of asphalt binder at high construction temperatures to ensure that binder is sufficiently fluid for pumping and mixing. The Brookfield rotational viscometer was used to determine all such viscosities. The standard ASTM method D4402 or AASHTO TP48 was adopted to determine the viscosity of neat and BG-modified binders. The viscosity readings were used to determine the improvement in PG grading and overall mixing and compaction temperatures. Each binder was tested for 135, 150, and 165°C.

### 3.4. Complex Shear Modulus (*G* ^*^)

Bohlin's dynamic shear rheometer (DSR) was used to conduct frequency sweep tests on neat and AC5 and BG-modified binders. Tests were conducted at temperatures of 1, 8, 15, 25, 34, 46, 52, and 58°C. The frequency sweep test was conducted to determine the complex shear modulus of binders. The tests were conducted within the viscoelastic stress and strain levels for a range of frequencies from 1 to 60 Hz at logarithmic increments.

### 3.5. Infrared (IR) Spectroscopy

FTIR analysis was performed with Thermo Scientific Nicolet iS10 FT-IR spectrometer in order to observe if the mixing process is homogeneous or not. As a matter of fact water can also be damaging for asphalt binder and water was used in the mixing process; hence FTIR was the best way to figure out if there are any water traces left in the mixture.

## 4. Results and Discussion

### 4.1. BG Mixing Process

This analysis helped determine the adequate time to evaporate the water from the mixture. It was found that with the increase of the BG dosage the time of evaporation increased. This issue was catered in establishing different times of mixing for different percentages of BG dosage, keeping few things constant, such as 200 g of asphalt and 130°C temperature using oil bath in a 600 mL beaker. The mixing times reported in Table 1 seem to be very less as compared to other polymer mixing times, especially when the mixing temperatures are also significantly higher, which results in less asphalt aging and less damage to the binder. This mixing procedure was also repeatable and consistent. It was also observed that the change of binder did not affect the mixing time, as the same time of 70 minutes was sufficient to evaporate the water from the PAC30 binder as was found for AC5 binder, which is a very unique finding of this study. This mixing procedure does not require excessive heating, which results in excessive energy loss and unaffordable process. This process utilized very less energy, which makes it not only energy conserving to reduce the cost of the binder preparation, but also environment friendly. Additionally, BG itself is not dangerous for human health and it is degradable as compared to other polymers. The limitation of the pan and beaker size restricted researchers to limited quantities; however, it is expected that it will be easier to evaporate the water from the mixture when this procedure is applied at a larger scale, which will be more effective in both ways: energy conservation and less aging of asphalt.

### 4.2. Viscosity

**Figure 1:**
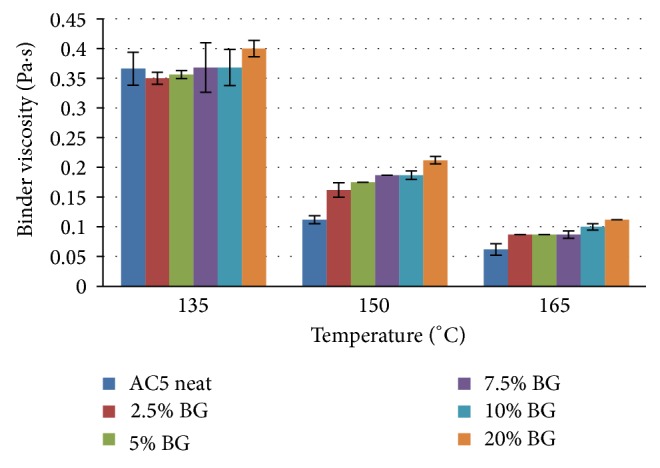
Viscosity of neat and BG-modified AC5 at various temperatures.

Figure 1 clearly shows that viscosity of the BG-modified AC5 binder did not change significantly at 135°C. This could be attributed to no aging effect during low temperature mixing process for a short time period. Moreover, there might be the liquefying effect of the BG and water solution. This result proved that the process of mixing is not only quick and easy, but also environment friendly, as the temperatures involved in this mixing process are very low, as compared to other polymer-modified asphalt mixing processes. On the other hand, viscosity of 20% BG-modified binder was increased up to 200%, at 150 and 165°C. This increase might be due to the stiffening effect of BG and aging due to extended mixing time.

The results of viscosity-temperature chart indicated that the mixing temperature ranges of HMA mixtures for AC5 neat and 10% BG-modified binder are 143 to 147°C and 148 to 158°C, respectively. Similarly, the compaction temperature ranges for AC5 neat and 10% BG-modified binder are 135 to 140°C and 139 to 144°C, respectively. It can be observed that average difference in mixing temperature is about 8°C and the average difference in compaction temperature is 4°C. Compaction is the main process in which most of the heating energy is consumed and environment is polluted, and the labor is also exposed to the emissions during this process. A study conducted by European Asphalt Pavement Association (EAPA) reveals that reduction of temperature by 10°C results in an emission reduction by 50% and a reduction in temperature by 11 to 12.5°C reduces the BSM (benzene soluble matter) fume emission by a factor of 2 [12, 13]. Moreover, another positive effect of the temperature reduction is a sustained improvement of the labor safety. This is a very important matter of concern in asphalt industry, especially in paving industry. The high emission of greenhouse gases is disastrous for environment and labor health. Lower compaction and mixing temperatures of BG modified binders as compared to polymer modified binders are remarkable achievements of the developed mixing procedure.

**Figure 2:**
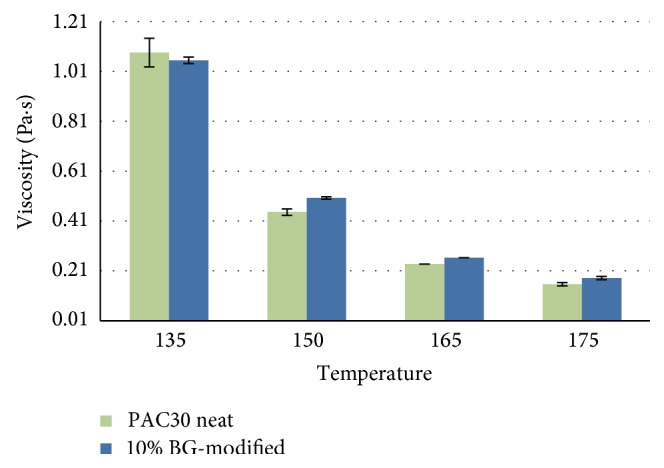
Viscosities of PAC30 and 10% BG-modified PAC30 at various temperatures.

Similar results were obtained for PAC30 binder (Figure 2). Interestingly, this binder was already modified with 4% SBS polymer and was further modified with 10% BG. The viscosity results showed no significant change in the binder viscosities.

### 4.3. Complex Shear Modulus

Rutting is a major problem in countries on equator or near equator. Warm to hot weather conditions are detrimental to asphalt pavements, especially if they are not modified. Hence we considered the superpave rutting parameter of the complex shear modulus (*G* ^*^/sin⁡*δ*) in order to compare the results of AC5 neat with 5%, 10%, and 20% and PAC30 neat with 10% modified binder, as shown in Tables 2 and 3. The data in the table indicates that the *G* ^*^/sin⁡*δ* increases with the increase of BG dosage showing an optimum value that is 10% BG, after which *G* ^*^/sin⁡*δ* decreases. 10% BG-modified binder exhibited average improvement of about 42% at 58°C. On the other hand, at 1°C the increase in *G* ^*^/sin⁡*δ* values is 36% to 81% from higher frequency to lower frequency, respectively. The improvements in rutting parameter as discussed above imply high resistance to permanent deformation or rutting. This improvement is encouraging and provides solid ground to explore other performance characteristics as well. Similar results were determined in PAC30 binder modification; the improvement was determined to be 23% on average at both temperatures.

### 4.4. Infrared (IR) Spectroscopy

**Figure 3:**
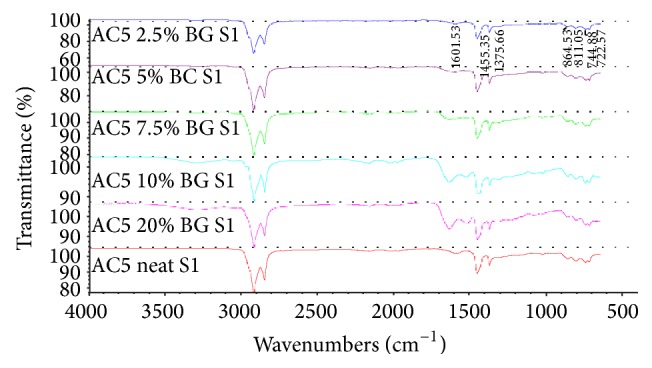
IR spectroscopy of neat and modified binders.

The infrared (IR) spectra of asphalt composite (AC5) show carbon-hydrogen stretching and bending frequencies characteristic of a hydrocarbon. IR peaks at 2850–2960 cm^−1^ can be attributed to the C–H symmetric and asymmetric stretch. The IR peaks at 1465 and 1375 cm^−1^ are characteristic of C–H bending vibrations (scissoring) while the peaks at 1150–1350 cm^−1^ most likely are due to C–H bending vibrations such as twisting and wagging. The peaks at 720–740 cm^−1^ are indicative of a C–H rocking vibrational mode. As one adds bone glue to the AC 5 sample, these same characteristic C–H vibrational modes of the asphalt composite are still present in the IR spectrum. However, as one increases the amount of bone glue to the sample, then the amount of water in the AC 5 sample also increases and this becomes observable in the IR spectrum. In particular, at the 10% level of bone glue in the AC 5 sample and at 20% bone glue, the O–H stretching frequency of water in the IR spectrum is observed at 3200–3400 cm^−1^, ~2100 cm^−1^, and 1640 cm^−1^. All other observable peaks in the IR spectrum appear to be unchanged. One may initially conclude that, with increasing bone glue and water addition to the AC 5 sample, the presence of water in the IR spectrum appears to be the major change. A longer reaction and evaporation time is warranted to remove the excess water seen in the sample (Figure 3).

## 5. Conclusions 

Based on the results and discussion the following conclusions and recommendations were drawn.

(1) The mixing procedure derived is cost effective and timesaving.
(2) The viscosity results indicate that mixing and especially compaction temperatures will not change in this modification, which makes it environment friendly and a better product in terms of labor health and safety.
(3) Improvement has been determined in *G* ^*^/sin⁡*δ* values, which is an indicator of rutting resistance improvement. This characteristic, especially, will help in warm climate countries like Southeast Asia.
(4) Use of another binder proved that the effect of the BG is not binder sensitive; rather it can improve any binder without affecting its mixing and compaction temperatures.
(5) FTIR analysis proved that the mixing procedure produced homogeneous mixing and no extra and abrupt peaks were observed in the spectrum. 10% and 20% mixtures exhibited some water traces, which can easily be removed by incrementing the mixing time to 2 to 3 minutes, as it was observed in PAC30 and 10% BG mixing.
(6) Further research on different percentages of BG in binder and further rheological studies in order to fully explore this modifying material are in process.

## Figures and Tables

**Table 1:** Mixing time and temperature.

BG dosage (%)	2.5	5	7.5	10	20
Time (min)	30	40	50	60	80
Temperature (°C)	130	130	130	130	130

**Table 2:** Comparison of *G* ^*^ at 1° and 58°.

AC5 N and BG binder *G* ^*^ at 1.57 Hz					
Temperature	AC5 N	5% BG	10% BG	20% BG	Diff.
1°C	3.4*e*7	3.5*e*7	5.9*e*7	3.1*e*7	74%
58°C	1.6*e*3	2.0*e*3	2.3*e*3	2.3*e*3	38%

**Table 3:** Comparison of *G* ^*^ at 1° and 58°.

PAC30 neat and BG at 1.57 Hz			
Temperature	PAC N	10% BG	Diff.
1°C	6.54*e*7	8.1*e*7	25%
58°C	5.8*e*3	7.02*e*7	21%
